# Correlation of the ultrasound thyroid imaging reporting and data system with cytology findings among patients in Uganda

**DOI:** 10.1186/s13044-023-00169-1

**Published:** 2023-09-01

**Authors:** Hamdi Mohamed Isse, Robert Lukande, Senai Goitom Sereke, Fualal Jane Odubu, Rita Nassanga, Samuel Bugeza

**Affiliations:** 1 Department of Radiology and Radiotherapy, College of Health Science, MakerereUniversity, Kampala, Uganda; 2 Department of Pathology, College of Health Science, MakerereUniversity, Kampala, Uganda; 3 Department of Surgery, College of Health Science, Makerere University, Kampala, Uganda

**Keywords:** TI-RADS, Ultrasound, Cytology, Fine needle aspiration, Thyroid nodule, Thyroid cancer

## Abstract

**Background:**

Ultrasonography is a noninvasive modality for the initial assessment of thyroid nodules. The American College of Radiology Thyroid Imaging Reporting and Data System (ACR TI-RADS) has demonstrated good performance in differentiating malignant thyroid nodules. However, the combination of ACR TI-RADS categories and cytology has not been studied extensively, in Uganda. The study aims to correlate ACR TI-RADS with cytology among patients referred for US-guided fine-needle aspiration at Mulago National Referral Hospital.

**Methods:**

This was a hospital-based cross-sectional study that recruited 132 patients with thyroid nodules. Spearman’s correlation was used to establish a relationship between TI-RADS and cytology findings. The diagnostic accuracy of TI-RADS was assessed using sensitivity, specificity, positive and negative predictive values, and positive and negative likelihood ratios.

**Results:**

Of 132 study participants, 90% (*n* = 117) were females, and the mean age was 41 ± 13 years. One hundred sixty-one thyroid nodules were analyzed. More than half of the thyroid nodules (54.7%, *n* = 87) were solid or almost solid, 96.9% (*n* = 154) were shaped wider than tall, 57.2% (*n* = 91) had smooth margins, 83.7% (*n* = 133) were hyperechoic or isoechoic, and 88.7% (*n* = 141) had no echogenic foci. TI-RADS 3 was the most common at 42.9% (*n* = 69). The proportions of malignancy for TI-RADS 4 and TI-RADS 5 were 73.3% and 85.7%, respectively. The correlation between ACR TI-RADS and the Bethesda system of thyroid classification scores was *r* = 0.577. The sensitivity, specificity, positive and negative predictive values, and positive and negative likelihood ratios of ACR TI-RADS were 90.9%, 98.5%, 90%, 99.3%, 62.3, and 0.1, respectively.

**Conclusion:**

We found that ACR TI-RADS classification is an appropriate and noninvasive method for assessing thyroid nodules in routine practice. It can safely reduce the number of unnecessary fine-needle aspiration in a significant proportion of benign thyroid lesions. Thyroid nodules classified as TI-RADS 3 should be followed routinely. ACR TI-RADS should be standardized as the screening tool in resource-limited areas.

## Background

Thyroid nodules (TNs) are a common thyroid disorder with a global prevalence ranging from 4–7% by palpation, 19–68% by ultrasound (US), and 8–65% by pathologic examination at autopsy [1, 2] This increase is thought to be related to early detection by high-resolution ultrasound and the discovery of subclinical TNs [3, 4] TNs can be classified as either benign or malignant. Most them are benign, and less than 5–10% are malignant [4]. In Africa, the prevalence of benign TNs is 89%, while that of malignant TNs stands at 11%, showing some variation from the expected global benign and malignant TNs percentages [5]. This could be due to the high prevalence of iodine deficiency goiter [6]. In Uganda, nodular thyroid disease is more common than diffuse thyroid disease, accounting for 82% of all patients referred with thyroid symptoms [7]. Furthermore, a study performed at Mulago National Referral Hospital (MNRH) found that 5% of the TNs evaluated were malignant, 18% were suspicious, and 75% were benign [7]. Hence, the need to identify suitable tools to assess the risk of malignancy in patients with TNs is crucial [8].

The differentiation of malignant and benign TNs of utmost importance in clinical evaluation since treatment is different for each type of nodule [9]. Ultrasound can be used to differentiate benign from malignant nodules based on certain characteristics [9]. To improve the diagnostic sensitivity and specificity of the ultrasound evaluation of TNs, the Thyroid Imaging Reporting and Data System (TI-RADS) was proposed [10]. The American College of Radiology Thyroid Imaging Reporting and Data Systems (ACR TI-RADS) is a 5-point classification system developed to determine the risk of cancer in TNs based on ultrasound characteristics. This system has been mainly used for TNs that are ≥ 1 cm. This system evaluates ultrasound features in five categories: composition, echogenicity, shape, margin, and echogenic foci; the nodule’s total points determine its risk level, which ranges from TI-RADS 1 (TR1) (benign) to TI-RADS 5 (TR5) (highly suspicious) [11]. None of these ultrasound features can be used in isolation to accurately differentiate benign from malignant TNs. Fine-needle aspiration (FNA) biopsy is the next critical step in the workup of a nodule after ultrasound identifies features that warrant biopsy [12]. The Bethesda System for Reporting Thyroid Cytopathology (TBSRTC) [13] was developed to standardize thyroid cytology diagnoses to convey the biopsy findings according to a classification system that provides clear management guidelines and the associated risk of malignancy [10]. ACR TI-RADS is relatively new [14] and has not been widely adopted for use in Uganda. Furthermore, the correlation of ACR TI-RADS with cytology has not been extensively studied in Uganda.

Age and sex correlate with the pathogenesis and increased prevalence of TNs [15, 16].

The incidence of nodules has been reported to be four times higher in women than in men [17]. This could be a result of hormonal influences of both estrogen and progesterone [18] Smoking, radiation exposure, pregnancy, multiparity, and abnormal body mass index ranges have also been identified as predisposing factors [19] Genetic factors, environmental influences, lifestyle, and access to medical care could be associated with variation in thyroid cancer incidence by geographic area and ethnicity [20]. Thyroid nodules are uncommonly cited in third world countries, where the disease is attributed to iodine deficiency disorders due to low salt consumption [21]. The study, therefore, sought to investigate the correlation of ACR TI-RADS with cytology among patients referred for US-guided FNA of thyroid nodules at MNRH.

## Methods

### Study design and setting

This was a hospital-based descriptive cross-sectional study conducted at the ultrasound unit of the Department of Radiology and Department of Pathology of MNRH, Kampala, Uganda, between November 2020 and March 2021. The radiology department provides wide imaging services, including US, computed tomography, plain radiography, and interventional radiology. It has eleven radiographers, five consultant radiologists, and four nurses. The department of pathology is situated at the School of Biomedical Science and serves the roles of teaching and research, as well as offering diagnostic histopathology/cytopathology and autopsy services. Most the patients referred for US-guided FNA are from the endocrine-surgical outpatient clinic at MNRH. The clinic runs every Wednesday from 8:00 AM to 2:00 PM and receives approximately 15–17 patients weekly.

### Study population

All consenting participants with thyroid nodules ≥ 1 cm on B-mode ultrasound scans were scheduled for US-guided FNA. All participants were at the age of 18 and above during the study period. Participants with prolonged bleeding time, extensively calcified nodules, emphysema, and clinical and laboratory features of thyrotoxicosis were excluded from the study.

### Sample size

The sample size was determined using Kish Leslie’s formula.

$$N=\frac{{Z}^{2}\alpha /2 p\left(1-p\right)}{{d}^{2}}$$

where:

**N** = desired sample size.

**Z** = Z score corresponding to 1.96 for 95% confidence level.

**p** = the estimated proportion of people with suspicious nodular thyroid sonography findings; 50% was used to obtain the maximum sample size

**d** = margin of error at the 95% level of significance, which is 0.05

Taking the prevalence of suspicious nodular thyroid sonographic findings as 50%, we obtained an approximate sample size of 385 participants.

The finite population correction formula was used to adjust the sample size before data collection since the accessible population was 200 patients for the duration of the study. The 200 participants were estimated by multiplying the number of FNA Referrals received per week [10] by 20 weeks (anticipated duration of the study).

Using the finite correction formula, the final adjusted sample size was 132 participants.

$$S=\frac{N}{{}^{1+N}\!\left/ \!{}_{Population size}\right.}=s=385/\left(1+385/200\right)=132$$

where:

S is the adjusted sample size.

Population size was the expected number of participants within the 5-month study period.

### Study procedure

All patients referred from the endocrine-surgical outpatient clinic with TNs in a B-mode US scan scheduled for US-guided FNA during the study period were screened for nodules using US. Those with TNs ≥ 1 cm in B-mode US and who consented were recruited into the study. Under the supervision of a radiologist, The US evaluation was performed on an SIUI machine, model Apogee 5300, manufactured January 2015 by Hamburg Germany. Ultrasound machine with high-frequency linear probes of 7.5 MHz for obese patients or large thyroid lesions, a 5 MHz transducer was used for greater penetration. US was performed with the patient in the supine position and the neck hyperextended, and the entire gland was examined. Hyperextension of the neck was obtained by placing a pillow under the shoulders. The neck was scanned in sagittal, transverse, and oblique sections to optimally visualize both lobes of the thyroid and isthmus. Color Doppler imaging was utilized. Imaging of the lower poles of the thyroid was obtained by making the patient swallow as this tends to raise the thyroid gland in the neck.

**Fig. 1:**
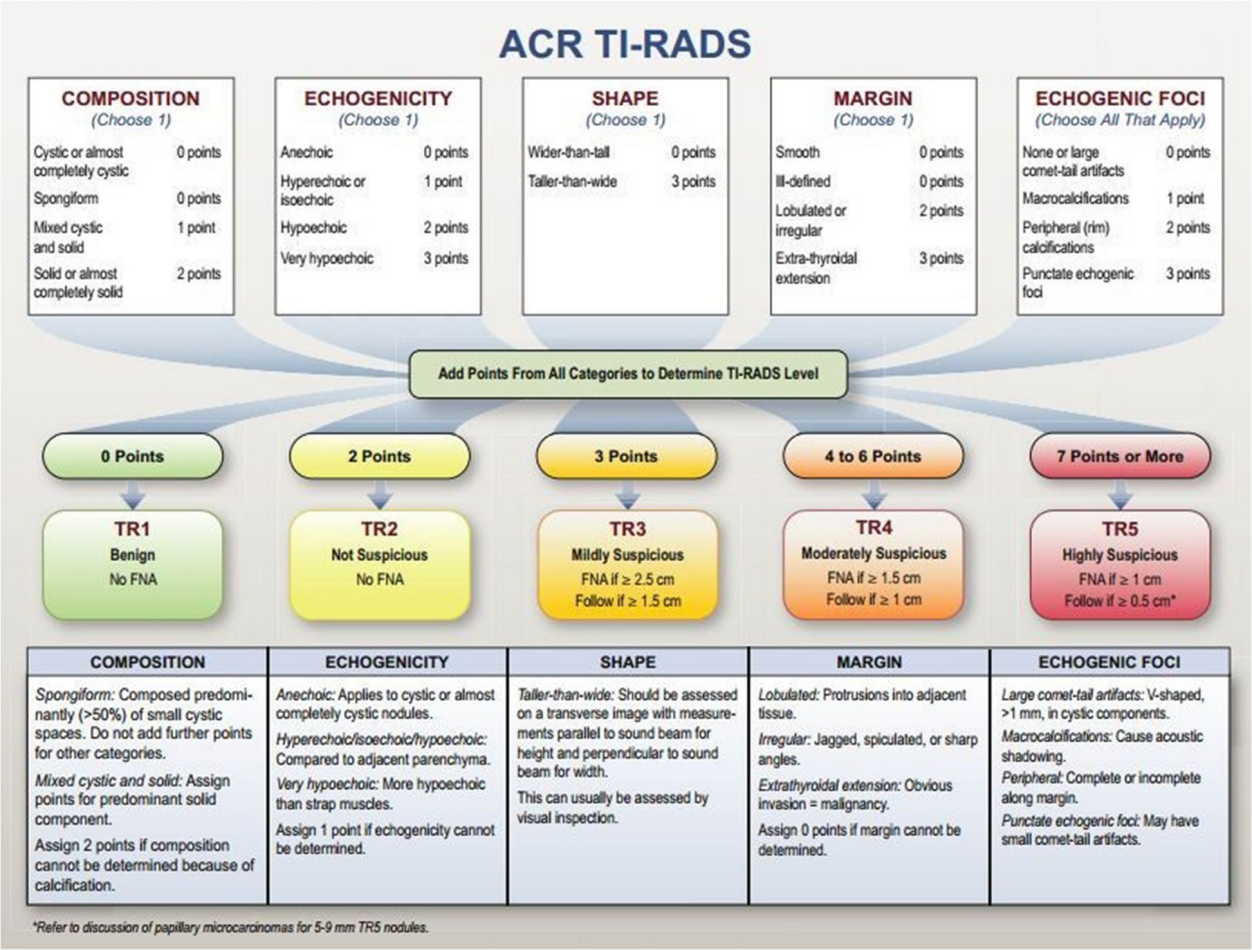
Nomenclature of categorization of thyroid nodule features per the five lexicon categories [22]

Thyroid nodules sonographic characteristics, such as composition, shape, echogenicity, margins and echogenic foci, were recorded, and points were assigned to each nodule for separate categories according to ACR TI-RADS guidelines [11]. The sum of the points in each category determined the TI-RADS level assigned to each nodule, with TR1 indicating 0 points; TR2 – 2 points; TR3 – 3 points; TR4 – 4–6 points; and TR5 – 7 or more points (Fig. 1). The final sonographic diagnosis was reached with the help of a consultant radiologist. The data obtained from the ACR point table were used to correlate with cytology results. A 23-gauge needle with a clear hub and clear syringe (5–10 ml) was used to obtain a sample from each nodule, and a maximum of two nodules were sampled per patient. The sample was gently expelled onto the surface of a labeled microscope slide from the needle tip. A smearing slide was then slid over the specimen, ensuring that both slides were smeared. One slide underwent wet fixation with alcohol, which was stained using the Papanicolaou method, while the other slide was air-dried at room temperature and stained with the Diff quick method. After this, an experienced pathologist evaluated all samples according to the TBSRTC [13]. The categories and their risk of malignancy were recorded as follows in (Table 1).

**Table 1:** The Bethesda system for reporting thyroid cytopathology

Categories	Description
I	Non-diagnostic
II	Benign
III	Atypia of undetermined significance (AUS)/follicular lesion of undetermined significance (FLUS),
IV	Follicular neoplasm (FN)/suspicious for follicular neoplasm (SFN)
V	Suspicious for malignancy (SM)
VI	Malignant

The ACR TI-RADS level, which ranges from TR1 (benign) to TR5 (high suspicion of malignancy), was also used to categorize the nodules. Coded US images were stored and printed.

### Statistical analysis

The data were entered into EpiData version 3.1 and then exported into Stata statistical software version 14 for analysis. To describe patient characteristics, categorical variables were summarized using frequencies and percentages, while continuous variables used the mean and standard deviation. While 132 patients were enrolled in the study, 29 of them had 2 or more nodules but only two nodules were biopsied, resulting in an analytic sample size of 161. Pairwise analysis was not performed to control for clustering because the patient identifiers were replaced with study numbers for the study purpose. The ACR TI-RADS classification criteria were used to classify nodules and then presented as frequencies and percentages. The difference in the proportions of the ACR TI-RADS sonographic criteria was tested using Fischer’s exact test. Spearman’s correlation coefficient was used to establish the correlation between ACRTI-RADS and cytology findings.

To determine the diagnostic effectiveness of ACR TI-RADS in characterizing thyroid nodules and predicting cytological findings, sensitivity, specificity, positive and negative predictive values, and positive and negative likelihood ratios with corresponding 95% confidence levels were calculated using the Bethesda system of thyroid classification as a gold standard. ACR TI-RADS was dichotomized by considering TR4 and TR5 as a positive screen for malignancy and TR1 to 3 as screen negative. The Bethesda System was also dichotomized by classifying 4 to 6 as malignancy and 1–3 as no malignancy.

## Results

Of the 132 study participants, the majority were females (90%, *n* = 117) with a mean age of 41 ± 13. Twenty-nine (22%) had 2 or more nodules, but only two nodules were biopsied, giving a total sample size of 161. Of these, 2 nodules of 2 patients were inadequate and were excluded from the final analysis. Most of the participants were from the central region of Uganda, 74 (56.9%) (Table 2).

**Table 2:** Sociodemographic characteristics of patients who underwent ultrasound-guided FNA in MNRH

Variables	Frequency *n* = 130	Percentage
**Gender**		
Male	13	10.0
Female	117	90.0
**Age in years**		
Mean ± SD	41 ± 13.0	
**Regions in Uganda**		
Central	74	56.9
Eastern	19	14.6
Western	21	16.2
Northern	16	12.3

*SD* standard deviation

**Fig. 2:**
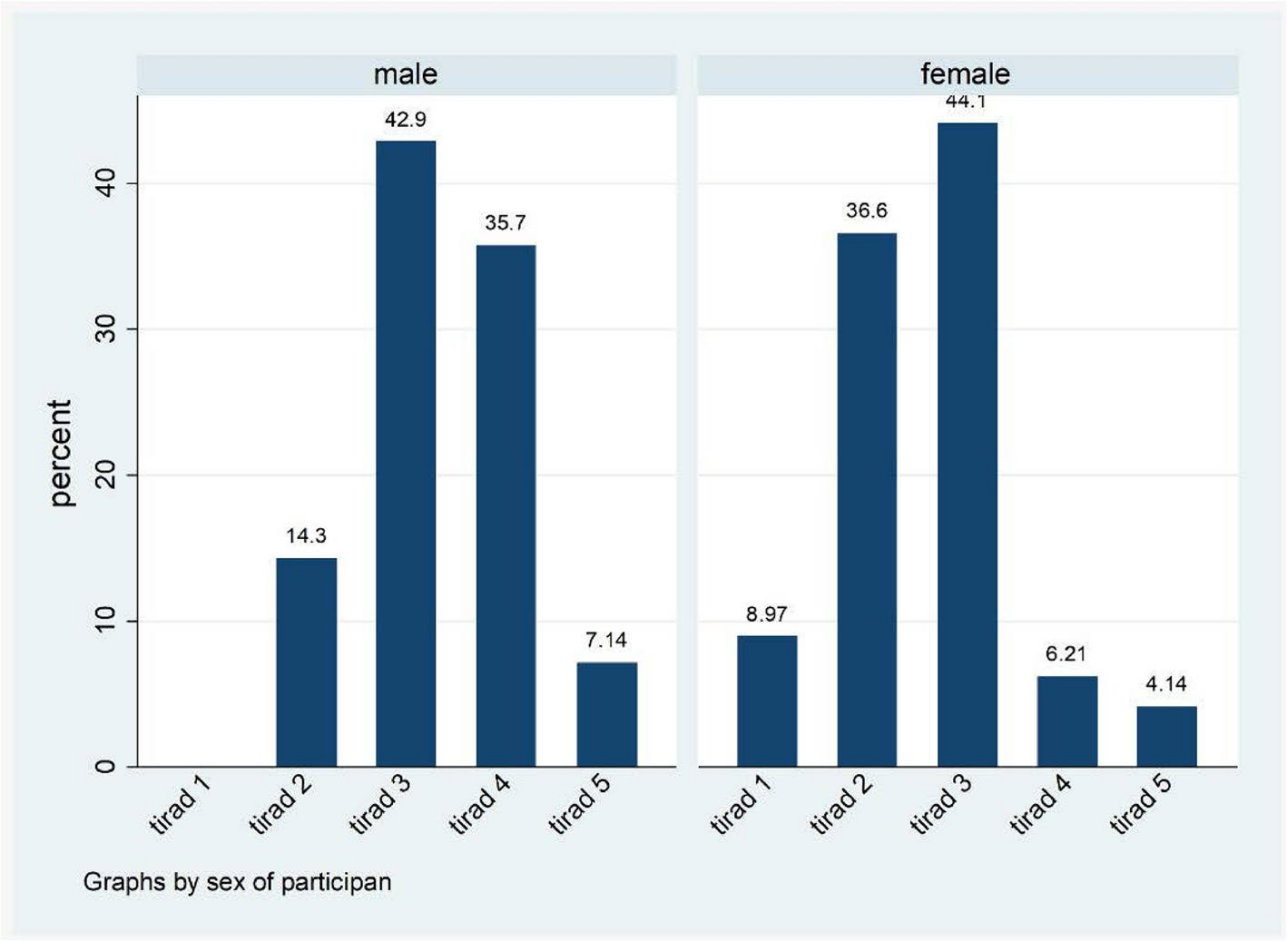
ACR TI-RADS classification of thyroid nodules stratified by sex

More than half of the nodules (87, 54.7%) had a composition that was mostly solid or almost solid, and 154 (96.9%) were shaped wider than tall. One hundred thirty-three (83.7%) of the nodules were hyperechoic/isoechoic, 60 (37.7%) had ill-defined margins, and 141 (88.7%) had no comet tail artifact or large comet tail artifacts (Table 3). Most of the nodules (69, 42.9%) were classified as TR3, followed by TR2 (56, 34.8%), 14 (8.7%) were classified as TR1 and 7 (4.3%) were classified as TR5. Furthermore, TR3 was the most frequently observed categorization in both males (42.9%) and females (44.1%) (Fig. 2).

**Table 3:** Sonographic appearance of thyroid nodules based on ACR TI-RADS

ACR-TIRADS based on	Frequency *n* = 159	Percentage
**Composition**		
Cystic/almost completely cystic	10	6.3
Spongiform	3	1.9
Mixed cystic and solid	59	37.1
Solid/ almost solid	87	54.7
**Shape**		
Wider than tall	154	96.9
Taller than wide	5	3.1
**Echogenicity**		
Anechoic	13	8.2
Hyperechoic or isoechoic	133	83.7
Hypoechoic	12	7.6
Very hypoechoic	1	0.6
**Margin**		
Smooth	91	57.2
Ill defined	60	37.7
Lobulated/ irregular	4	2.5
Extra thyroid extension	4	2.5
**Echogenic foci**		
None/ large comet tail artifact	141	88.7
Macro calcification	12	7.6
Peripheral calcifications	1	0.6
Punctate echogenic foci	5	3.1

Two (1.2%) of the nodules classified as Bethesda 1 and 141 (85.5%) classified as Bethesda II were considered benign, and 18 (11.2%) nodules classified as Bethesda 4–6 were considered malignant. Six nodules (3.7%) that were categorized as TR5 truly turned out to be malignant according to the Bethesda classification (Bethesda 6). Similarly, most of the nodules categorized as TR4 were suspicious for malignancy according to the Bethesda classification (Bethesda 5). One out of the 141 nodules characterized as TR5 on ultrasound was Riedel’s thyroiditis (Bethesda 2).

Moreover, one [1] out of the 69 nodules categorized as TR3 was classified as suspicious for follicular neoplasm (Bethesda classification 4). When comparing the ACR TI-RADS classification with the TBSRTC, the proportion of malignancy for TR1, 2, 3, 4, and 5 was 0, 0, 1.4, 73.3, and 85.7%, respectively. The risk of malignancy was determined by dividing total Bethesda scores from 4–6 by the total ACR TI-RADS level (Table 4). There was a moderate correlation between ACR TI-RADS and the Bethesda system of thyroid classification scores (*r* = 0.577), and this correlation was statistically significant (p < 0.001).

**Table 4:** Correlation of ACR TI-RADS and Bethesda system of thyroid classification

	**BETHESDA system of thyroid classification**							
**ACR-TIRADS categorization**	**1**	**2**	**3**	**4**	**5**	**6**	**Total n (%)**	Proportion of malignancy (%)
TIRADS 1	1	13	0	0	0	0	14 (8.7)	0
TIRADS 2	1	55	0	0	0	0	56 (34.8)	0
TIRADS 3	0	68	0	1	0	0	69 (42.9)	1.4
TIRADS 4	0	4	0	2	8	1	15 (9.3)	73.3
TIRADS 5	0	1	0	0	0	6	7 (4.3)	85.7
**Total n (%)**	2 (1.2)	141(85.5)	0(0)	3(1.8)	8(4.9)	7(4.3)	161	

The sensitivity and specificity of ACR TI-RADS to detect malignancy were 94.4% with a 95% CI of 0.944 (0.8–1.0) and 96.5% with a 95% CI of 0.965 (0.9–0.99), respectively. The positive (PPV) and negative (NPV) predictive values corresponding to the above sensitivity and specificity were 77.3% and 99.3%, respectively, while the positive and negative likelihood ratios were 27 and 0.06, respectively (Table 5). Hypoechoic echogenicity and solid composition combined with hypoechoic echogenicity showed the highest sensitivity to detect malignancy of 54.6% and 57.1%, respectively. All the parameters used in the classification of ACR TI-RADS except for composition were statistically significantly associated with cytology results, *p* < 0.005. This significant association was noted for nodules that were either solid and hypoechoic or solid and hyperechoic (Table 6).

**Table 5:** 2 × 2 ACR TI-RADS categorization of thyroid nodules and the gold standard

		**Gold Standard**		
		**Malignant**	**Benign**	**Total**
**ACR TI-RADS**	**Positive**	17	5	22
	**Negative**	1	138	139
	**Total**	18	143	161

Sensitivity = (17/18)*100 = 94.4% with 95% CI of 0.944 (0.8–1.0); Specificity = (137/143)*100 = 96.5% with 95% CI of 0.965 (0.9–1.0). PPV = (17/22)*100 = 77.3%; NPV = (138/139)*100 = 99.3%; LR Positive = 94.4/(100–96.5) = 27; LR Negative = (100–94.4)/96.5 = 0.06

**Table 6:** Distribution of sonographic features used in the ACR TI-RADS categorization of thyroid nodules across the FNA results

ACR-TIRAD and its classification parameters	Malignancy	Benign	*p*-value*
	**(%)**	**(%)**	
**Composition**			
Solid ( +)			
Non solid (-)	21.8	1.4	0.217
**Shape**			
Taller than wide ( +)			
Wider than tall (-)	10	0.7	0.001
**Echogenicity**			
Hypoechoic ( +)			
Anechoic/hyper echoic/			
Isoechoic (-)	54.6	0.7	< 0.001
**Margins**			
Lobulated/irregular/extra			
Thyroid extension ( +)			
Smooth/ ill defined (-)	31.8	0.7	< 0.001
**Echogenic foci**			
Punctate echogenic foci/			
Peripheral calcification ( +)			
None or large comet tail			
Artifact/ macro calcification (-)	27.3	0	< 0.001
**Echogenicity & composition combined**			
Solid& hypoechoic nodule ( +)			
Solid & hyperechoic nodule (-)	57.1	1.5	< 0.001

^*^p-values obtained from running Fisher’s exact test

**Fig. 3:**
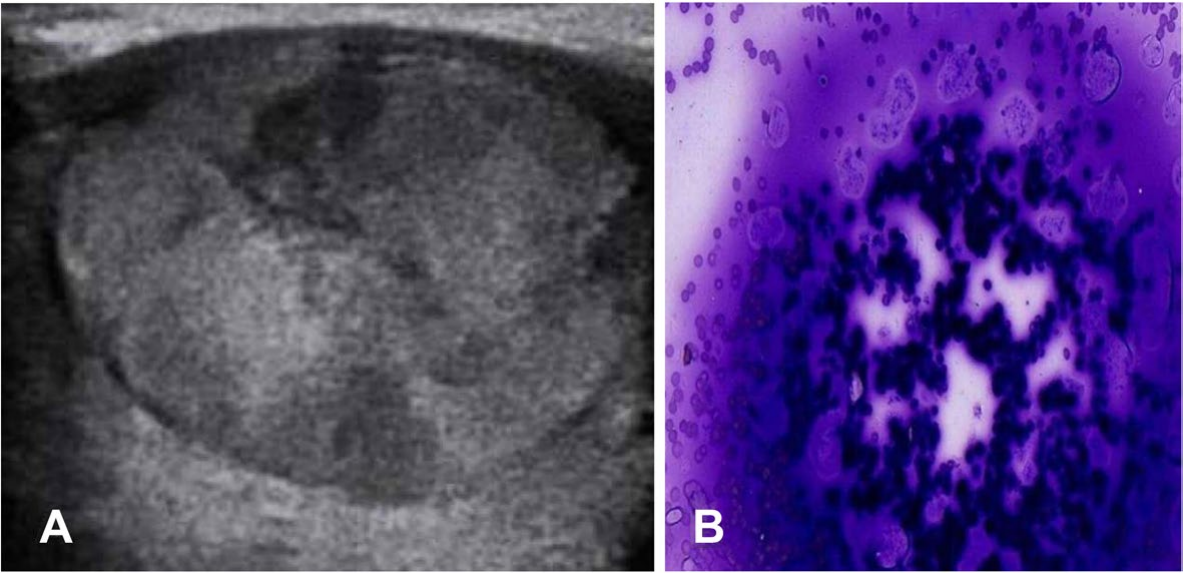
**A** Transverse sonogram of a well-defined wider than taller isoechoic solid nodule in the upper outer quadrant of the left lobe in 24-year-old women. It was classified as solid (composition score of 2), With 1 more point for iso-echogenicity and none in other categories, its total point was 3 (TR3). **B** Cytology result showing abundant thick colloid benign follicular cells (diff-quick, × 40) (category II bethesda system)

**Fig. 4:**
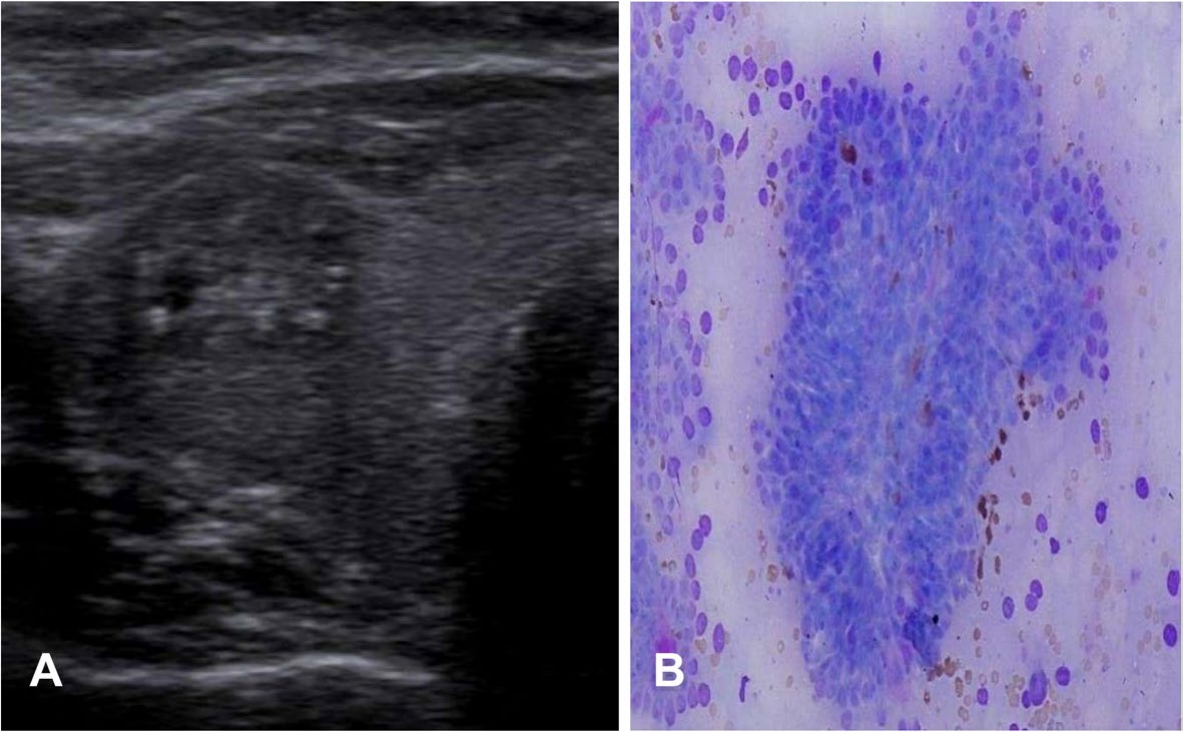
**A** A solid hypoechoic nodule with punctate calcification in an 63-year-old man. The nodule received 2 points for composition, 2 for being hypoechoic, and 3 for punctate calcification, for a total of 7 (TR5). **B** shows papillae lined by cells with marked enlarged, and crowded/overlapped nuclei (Diff-Quick,40) (papillary carcinoma-Category VI Bethesda system)

An US images of the TR3 and TR5 nodules with their corresponding categories of II (Benign follicular cells) and VI Bethesda system (papillary carcinoma) were shown in Figs. 3 and 4.

## Discussion

This study sought to determine the correlation of ACR TI-RADS with TBSRTC among patients attending MNRH. We found high sensitivity and specificity of ACR TI-RADS to detect malignancy and a strong positive correlation between ACR TI-RADS and TBSRTC. Moreover, the majority of the nodules were classified as TR3 based on sonographic appearance, and the majority of the TNs referred for FNA were solid/almost solid, wider than tall, hyperechoic or isoechoic, had a smooth margin, and had no echogenic foci.

The study observed that the majority of the patients were females with an average age of early forties, which was consistent with other studies performed in Uganda [7], India [23], and Saudi Arabia [24] that reported a similar female to male ratio and average age. This can be explained by the fact that females are more prone to symptomatic thyroid nodular disease as a result of hormonal influences of estrogen and progesterone [25].

The sonographic appearance of thyroid nodules based on ACR TI-RADS demonstrated the majority to be solid or almost solid, wider than tall in appearance, smooth margins, hyperechoic or isoechoic and having no echogenic foci. These findings are consistent with features often seen on benign nodules. A cross-sectional study performed in India among 104 patients showed similar sonographic features on the thyroid gland nodules analyzed [26]**.** Another comparable study in the Philippines showed that the most frequent characteristics of nodules on US were solid in composition, isoechoic to hyperechoic, and wider than tall; however, few had well-circumscribed margins [27].

Of all the 161 nodules categorized based on ACR TI-RADS, approximately 42.9% were classified as TR3, followed by TR2, only less than 10% were TR4 and TR1, and the least common was TR5. Our findings and those from previous studies present a heterogeneous picture of ACR TI-RADS classification. For instance, a French study [28] observed a similar finding to ours, with the majority having TR3 nodules. However, a similar study from India reported TR2 as the most prevailing category [17] Regardless of these differences in ACR TI-RADS categorization, the majority of the nodules in these studies were still classified as benign nodules. Our study also observed that the risk of malignancy increased as the level of ACR TI-RADS categorization increased, from 0% in TR1 and TR2 to 73% and 86% in TR4 and TR5, respectively. These findings were consistent with studies from France [28] and South Korea [9].

Our study demonstrated a significant strong positive correlation between the ACR TI-RADS scoring of thyroid nodules and Bethesda System classification scores. Other studies have also found a strong correlation between the two methods [29]. In addition, the study found a high sensitivity (94%) and specificity (96%) with excellent PPV (77%) and MPV (99%). The ACR TI-RADS showed a positive likelihood ratio of 27 and a negative likelihood ratio of 0.06, which implies that ACR TI-RADS is moderately good at ruling in benign or malignant thyroid nodules and ruling out benign or malignant ones, respectively. The ACR TI-RADS was also statistically significantly associated with cytology results (*p* < 0.001). Compared to other studies, a study in India revealed a comparable sensitivity and specificity of over 90% [17]. The study, however, reported a lower PPV and similar NPV [17]. The low PPV in the Indian study is possibly due to a higher prevalence of thyroid malignancy compared to a lower prevalence in Uganda.

This study found hypoechogenicity on ultrasound to be a predictor of malignancy with a reduced sensitivity of 54.6% but increased specificity and positive and negative predictive values of over 90%. Similar studies that were performed in Sri Lanka [30] and India [31] also demonstrated similar findings. Furthermore, irregular margins as well as taller than wider shapes are also predictors of malignancy, albeit with a low sensitivity of 31.8%, while the specificity and positive and negative predictive values were almost 90%. Comparable findings were reported by the same study from India [31], who observed that poorly defined irregular margins had equally low sensitivity but high specificity and positive predictive value. These findings are also similar to a study reported by Jabar et al. that showed irregular margins and taller than wider appearances had low sensitivity and PPV and high specificity and NPV [32]. On the other hand, the French study [28] showed that taller than wide as a predictor for malignancy had a very low sensitivity, good specificity, and high PPV and NPV.

The study had some limitations. Analysis of intra-rater variability could not be conducted since the study did not have a control group, and patients could not be used as their controls due to the limited number of biopsies that can be tolerated by patients. Also, some of the study limitations were short sample size and dichotomization of the Bethesda System.

Since patients with only ultrasound scan results were included, these introduced selection bias. The use of two biopsies from a single participant may have introduced a clustering effect, which may have biased our findings.

## Conclusion

We found that ACR TI-RADS is an appropriate and noninvasive method for assessing thyroid nodules in routine practice. This scoring system can safely reduce the number of unnecessary biopsies in a significant proportion of patients with benign thyroid lesions. Thyroid nodules classified as TR3 should be followed routinely. We recommend sonographic categorization of thyroid nodules using ACR TI-RADS in Uganda to create uniformity in reporting and easy guidance of appropriate biopsy.

## Data Availability

The datasets used and/or analyzed during the current study are available from the corresponding author on reasonable request.

## Abbreviation

ACR: American College of Radiology
FNA: Fine needle aspiration
MNRH: Mulago National Referral Hospital
NPV: Negative predictive value
PPV: Positive predictive value
TBSRTC: The Bethesda System for Reporting Thyroid Cytopathology
TNs: Thyroid nodules
TI-RADS: Thyroid Imaging Reporting and Data System
TR: TI-RADS
US: Ultrasonography
